# Mast Cell Targeted Chimeric Toxin Can Be Developed as an Adjunctive Therapy in Colon Cancer Treatment

**DOI:** 10.3390/toxins8030071

**Published:** 2016-03-11

**Authors:** Shan Wang, Linmei Li, Renren Shi, Xueting Liu, Junyan Zhang, Zehong Zou, Zhuofang Hao, Ailin Tao

**Affiliations:** 1 The Second Affiliated Hospital of Guangzhou Medical University, Guangdong Provincial Key Laboratory of Allergy & Clinical Immunology, The State Key Clinical Specialty in Allergy, The State Key Laboratory of Respiratory Disease; Guangzhou 510260, China; april0860@sina.cn (S.W.); lilinmei_sysu@163.com (L.L.); renrenshi1988@163.com (R.S.); xiaoyeliu1984@sina.com (X.L.); allergy_zhang@sina.cn (J.Z.); zouzehong123@126.com (Z.Z.); 2 The Second Affiliated Hospital of Guangzhou Medical University, Guangzhou 510260, China; zhuofanghao@sina.com

**Keywords:** colon cancer, tumor microenvironment, chimeric toxin, Fcε-PE40, mast cell

## Abstract

The association of colitis with colorectal cancer has become increasingly clear with mast cells being identified as important inflammatory cells in the process. In view of the relationship between mast cells and cancer, we studied the effect and mechanisms of mast cells in the development of colon cancer. Functional and mechanistic insights were gained from *ex vivo* and *in vivo* studies of cell interactions between mast cells and CT26 cells. Further evidence was reversely obtained in studies of mast cell targeted Fcε-PE40 chimeric toxin. Experiments revealed mast cells could induce colon tumor cell proliferation and invasion. Cancer progression was found to be related to the density of mast cells in colonic submucosa. The activation of MAPK, Rho-GTPase, and STAT pathways in colon cancer cells was triggered by mast cells during cell-to-cell interaction. Lastly, using an Fcε-PE40 chimeric toxin we constructed, we confirmed the promoting effect of mast cells in development of colon cancer. Mast cells are a promoting factor of colon cancer and thus also a potential therapeutic target. The Fcε-PE40 chimeric toxin targeting mast cells could effectively prevent colon cancer *in vitro* and *in vivo*. Consequently, these data may demonstrate a novel immunotherapeutic approach for the treatment of tumors.

## 1. Introduction

Tumor development depends on the balance of tumor promoting and anti-tumor effects *in vivo*. Cancer regulation comes from the tumor cell itself and the surrounding microenvironment. Immune cells are an important component of the tumor microenvironment. The most common immune cells in the tumor microenvironment are T cells and tumor-associated macrophages (ATMs). T cells in the tumor microenvironment include cytotoxic T cells and helper T cells that can have either a tumor suppressing or tumor promoting role [1]. ATMs are the most numerous of the inflammatory cells in tumor stroma and can secrete a variety of proinflammatory cytokines, growth factors and chemokines. ATMs, together with dendritic cells, mast cells and other immune cells, form the inflammatory microenvironment around the tumor [2]. The relationship between inflammation and tumors has been much discussed and it has become evident that an inflammatory microenvironment is an essential component of all tumors, including some in which a direct causal relationship with inflammation has not yet been proven [3,4].

Current research suggests that an inflammatory microenvironment promotes tumor development in the following ways: (I) Cytokines that stimulate tumor cell proliferation and survival, including epidermal growth factor (EGF), platelet-derived growth factor (PDGF), hepatocyte growth factor (HGF) and basic fibroblast growth factor (bFGF) are present in the inflammatory microenvironment and shift the balance between neoplastic proliferation and apoptosis [5]. Tumor development depends on the increase of cell proliferation and the reduction of cell death. The MAPK pathway is one of the most important signal transduction pathways in cells, mediating the cell stress reaction to proliferation, differentiation and cell death, *etc*. Studies have revealed that many inflammatory cytokines can activate protein kinases in the MAPK signaling pathway such as ERK, p38MAPK and JNK and play roles in tumor promotion [6,7,8,9]. (II) Inflammatory cells can produce proteases (such as metal protease, plasmin, *etc.*) to help eliminate extra protein in the cell matrix, leading to tumor cell basement membrane decomposition [10,11]. Inflammatory cytokines (such as IL-1, IL-6, *etc*.) can also activate Rho/ROCK signaling pathways within tumor cells and result in increased cell mobility [12]. In addition, research has shown that tumor cells in an inflammatory environment *in vivo* expressed decreasing amounts of intercellular adhesion proteins such as E-cadherin, β-catenin, *etc.* that facilitate tumor cell detachment from the matrix and migration [13]. (III) Inflammatory cells in the tumor microenvironment can produce large quantities of angiogenic factors and growth factors such as vascular endothelial growth factor (vEGF), TNF a, IL 8 and bFGF. They can also secret cytokines to make tumor cells express angiogenic factors and growth factors. These factors promote angiogenesis and lymphangiogenesis in the tumor microenvironment that leads to increased blood supply to the tumor and metastasis [14,15]. (IV) In addition, the inflammatory microenvironment could also suppress the defense response of immune cells around the tumor by reducing their cytotoxicity through immune-regulatory factors that allow tumor cells to escape [2,16]. In short, inflammatory cytokines in the tumor microenvironment can act on tumor cells by activating different downstream effectors to promote the proliferation, migration and differentiation of tumor cells. As multiple-gene regulation transcription factors, NF-kB and AP-1 are often considered the intersections of intracellular pathways started by inflammatory cytokines [17,18]. Because signal transducers and activators of transcription (STATs) can rapidly transmit cytokine signals from the plasma membrane to the nucleus without the involvement of a second-messenger signaling cascade, members of STATs have been found to be involved in the above four aspects of the effects of inflammation on tumor cells [19,20,21].

Mast cells (MC) are one of the earliest immune cells recruited during tumorigenesis [22]. In addition to their key role in allergy, mast cells are also a crucial immune cell able to release cytokines in the inflammatory microenvironment that can affect tumor growth. Mast cells are capable of secreting a variety of bioactive mediators stored inside particles in their cytoplasm. These mediators mainly include proteases (such as tryptase, chymase), cytokines, chemokines, and angiogenic factors [23,24]. MCs release their immune mediators by degranulation after sensitization. MC degranulation can be activated by different pathways. In addition to the classical pathway mediated by IgE binding to mast cell surface FcεRI receptors, MC can be induced to degranulate directly by activated C3a and C5a produced in the inflammatory microenvironment [25]. Furthermore, MCs can also slowly release immune mediators through “piecemeal degranulation” [26,27] that can occur by engagement of the c-Kit receptor or other pattern recognition receptors on the mast cell surface. Activation of IgE-independent alternative pathways has been frequently observed in mast cells infiltrating tumors [28,29]. Because of the diversity of the bioactive mediators released, MCs have been found to be attributed alternatively to both tumor rejection and tumor promotion [30]. Depending on the tumor setting, mast cells can directly influence tumor cell proliferation and invasion through the release of proangiogenic factors and matrix metalloproteinase [31,32]. MCs can also release immunosuppressive cytokines like interleukin-10, which can help tumor development by organizing its microenvironment or modulating immune responses [33]. In addition, the mediators released by MCs are crucial for the recruitment of other inflammatory cells such as macrophages, neutrophils, and eosinophils in tissues [34]. Furthermore, MC were observed to inhibit tumor development, which was attributed to the release of pro-inflammatory cytokines and proteases such as TNF-α and tryptase [35]. Among all the immune mediators released by MCs, histamine is one that has attracted attention in the context of early cancer therapy. Investigators of a trial that treated colon cancer patients with the histamine antagonist Cimetidine for seven days perioperatively reported an increased survival benefit from the Cimetidine treatment [36] and another trial in which Cimetidine was studied for symptomatic relief in colon cancer patients reported a similar survival benefit [37,38]. To sum up, the potential role of mast cells in cancer biology provides a new target for tumor therapy.

In the past few years, the correlation between mast cell infiltration and cancer prognosis has been described in different human tumors *in vivo* [39,40]. For example, MC infiltration in prostate tumors is an independent prognostic factor and predictor of poor outcome in these patients [41]. MC tryptase can be detected in the peripheral blood of pancreatic cancer patients [42]. In hepatocellular carcinoma, higher peritumoral MC density was associated with poorer clinical outcomes and an increased probability of early recurrence [43]. As a specific marker of mast cells, c-kit expression has been found to be a poor prognostic indicator in phyllodes tumors of the breast [44]. In colorectal cancer, high MC density has been found to be positively correlated with tumor angiogenesis [45,46] and could be a predictive marker of poor clinical outcome in these patients [47].

Although some signaling molecules in the pathways of mast cells have been reported to promote tumor cells, there is no comprehensive description of the pathways and mechanisms that mast cells utilize in cancer development. Therefore, in this article and using colon cancer as a model, we will evaluate the role of mast cells in promoting cancer development *in vivo* and *in vitro*. We will analyze the mechanisms used by mast cells for cell proliferation, cell migration and angiogenesis of colon cancer. In addition, the participation of the MAPK pathway, Rho/ROCK pathway and STATs in these processes will also be systematically designed and analyzed. If the role of mast cells in the development of colon cancer is confirmed, mast cell targeted therapy could then be applied as an adjuvant treatment for colon cancer. Accordingly, we created an Fcε-PE40 chimeric toxin [48,49] that can kill mast cells through the specific binding of FcεRI. The activity of this toxin in our experiments further shows the importance of mast cells in promoting colon cancer.

## 2. Results

### 2.1. Mast Cell Infiltration Is Associated with Colon Cancer Development and Distant Metastasis

**Figure 1:**
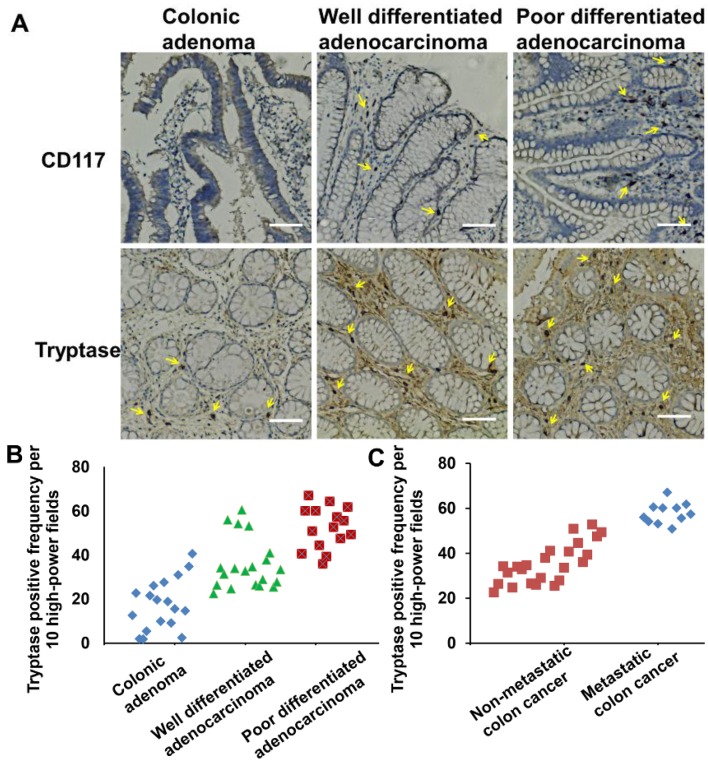
Mast cell infiltration relates with colon cancer development. (**A**) Immunohistochemistry-stained pictures of CD117 and tryptase show mast cells infiltration in different colonic tumor tissues. Magnification, ×400. Scale bar, 100 μm. (**B**) Mast cell counts carried out on tryptase Immunohistochemistry-stained colonic tumor sections in per 400× field. Tumor tissues are characterized by colonic adenoma, well differentiated adenocarcinoma, and poor differentiated adenocarcinoma. (**C**) Mast cell counts carried out on tryptase immunohistochemistry-stained colonic tumor sections. Tumor tissues are characterized by non-metastatic colon cancer and metastatic colon cancer. Arrows indicate mast cells.

To understand the relationship between mast cell infiltration and colon cancer development, we assessed the quantity of mast cells among different pathological colonic tissues. In our study, mast cells were identified by immunohistological detection as c-kit^+^ and tryptase^+^ (Figure 1A) [50,51]. Colon tissues were obtained at various stages of cancer development and grouped by pathology: colonic polyps (*N* = 18), well-differentiated colonic adenocarcinoma (*N* = 20), and poorly-differentiated colonic adenocarcinoma (*N* = 15). Data were analyzed from colonic submucosa. The mast cell staining index scores of the three groups were 18, 36 and 52, respectively. From the data analysis, mast cell infiltration in colorectal cancer was significantly higher when compared with colonic polyps (*p* < 0.05). Additionally, there was more mast cell infiltration in the poorly-differentiated colon cancer tissues than in the well-differentiated colon cancer tissues (*p* < 0.05) (Figure 1B).

Next, in order to evaluate the relationship between mast cell infiltration and distant metastases of colon cancer cells [52], we obtained colon cancer tissues and divided them into two groups: one group without distant metastasis (*N* = 48) and the other with either lymph node metastasis or distant organ metastasis (*N* = 11). Data were obtained by the same method as above. The mast cell staining index scores of the two groups were 35 and 58, respectively (Figure 1C). According to the statistical analysis of the data Figure 1C, there was a significantly higher level of mast cell infiltration in the colon tissues of patients that had distant metastases (*p* < 0.05).

These data show that MC infiltration significantly increases upon tumor progression from polyps to adenocarcinoma and also shows a positive correlation with the presence of distant metastases.

### 2.2. Mast Cells Induced from Bone Marrow Cells Can Manifest “Piecemeal Degranulation” and Promote Colon Cancer Cell Proliferation and Migration in Vitro

**Figure 2:**
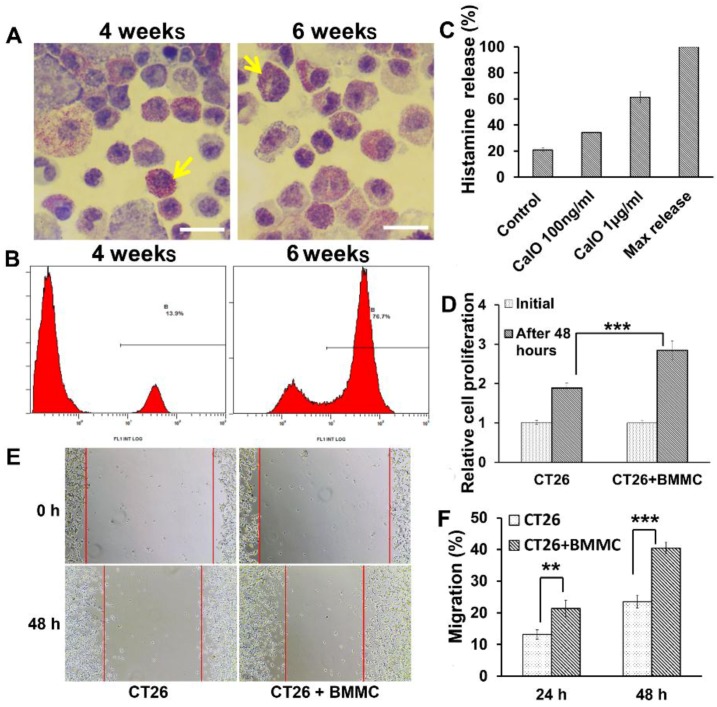
Piecemeal degranulation of mast cells can promote colon cancer cell proliferation and migration. (**A**) Toluidine blue staining of bone marrow-derived mast cells induced for 4 weeks and 6 weeks. Arrows indicate mature mast cells enriching with granules. Magnification, ×1000, Scale bar, 20 μm. (**B**) Histogram of bone marrow-derived mast cells (BMMCs) induced for 4 weeks and 6 weeks. Mast cells were identified by flow cytometry as CD117+ cells which were 13.9% at 4 weeks and 76.7% at 6 weeks. (**C**) Histamine release analysis shows bone marrow-derived mast cell “piecemeal degranulation” without stimulation and Calcium Ionophore (CaIO) makes bone marrow-derived mast cells degranulation with a dose-dependent way. (**D**) Quantification of CT26 cells proliferation cultured with or without BMMCs after 48 h by MTS. (**E**,**F**) The wound healing assay showed different cell migration in CT26 cells and CT26 cells co-cultured with BMMCs. (**E**) Representative images were taken at different time points. (**F**) Quantification of CT26 cells migration by measuring the wound width. The amount of motility was expressed as a percent of migration at the time point 0. ** *p* < 0.01; and *** *p* < 0.001.

To further analyze the functional role of mast cells in colon cancer development *in vitro*, we produced bone marrow-derived mast cells from mouse bone marrow cells co-cultured with IL-3 and SCF [53]. Cells were cultured for up to six weeks and then characterized by morphology and protein markers specifically expressed in mast cells [54]. Mature mast cells contain basophilic granules that can be identified as purple red granules by toluidine blue staining. We smeared bone-marrow-derived mast cells onto glass slides and stained them with toluidine blue. Most cells contained abundant purple red basophilic granules indicating that differentiation to mast cells had occurred (Figure 2A). Flow cytometry were used to detect the expression of CD117 on the surface of bone marrow-derived mast cells induced after two and six weeks, respectively. Figure 2B shows a significantly increased number of cells expressing c-kit at six weeks compared to cells at four weeks (76.7%, 13.9%, *p* < 0.01). Thus, we considered the bone marrow-derived mast cells (BMMCs) to be mature after a six-week induction period at which time they were used in our experiments.

Since we were able to produce mature mast cells, we wondered how much “piecemeal degranulation” they had without IgE involvement. The release of histamine has been used to evaluate the degranulation of mast cells. BMMCs without stimulus released up to 21% of their maximum histamine release (Figure 2C) indicating that immune mediators released from BMMCs during “piecemeal degranulation” could reach 21% of their maximum.

To assess the effects of mast cells on tumor growth, the colon cancer cell line CT26 was co-cultured with the BMMCs obtained above for 24 and 48 h. We first used an MTS assay to examine the effect of mast cells on colon cancer proliferation. As shown in Figure 2D, the rate of CT26 cell proliferation was increased in the group co-cultured with mast cells compared with the control group at 48 h (*p* < 0.001), indicating that mast cells can significantly promote the proliferation of colon cancer cells. Furthermore, we used a wound-healing assay to examine the effect of mast cells on the migration of colon cancer cells. As shown in Figure 2E, when compared with the control group, the group co-cultured with mast cells exhibited a considerably quicker migration. Quantification of wound closure showed that the CT26 control group closed 13.2% of the wound while CT26 cells co-cultured with mast cells closed 21.3% of the wound after 24 h (*p* < 0.01). Even more remarkable, at 48 h the CT26 control group closed 23.4% of the wound while the CT26 cells co-cultured with mast cells closed 40.4% (*p* < 0.001) (Figure 2F). Based on the above findings, mast cells can significantly promote the proliferation and invasion of colon cancer cells *in vitro*.

### 2.3. Mast Cells Can Up-Regulate the Expression of RhoA and VEGFc in Colon Cancer Cells to Promote Cancer Invasion and Angiogenesis

**Figure 3:**
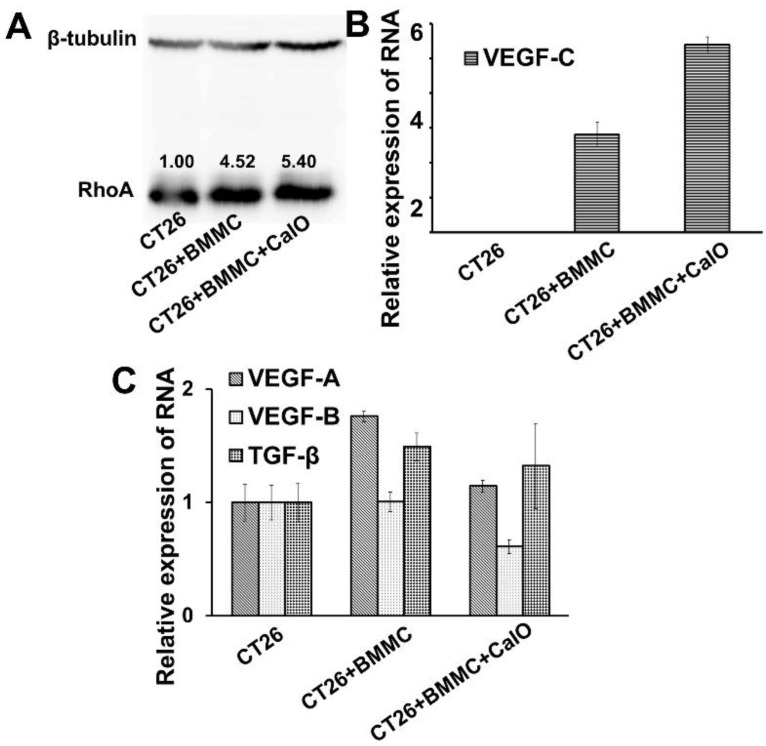
Mast cells up-regulate the expression of RhoA and VEGFc in colon cancer cells. (**A**) Western blotting analysis of the expression levels of RhoA in CT26 cells with different stimulation conditions. (**B**,**C**) RT-PCR analysis detected the VEGF-A/B/C and TGF-β expression in CT26 cells with or without BMMCs co-cultured.

Since we confirmed that mast cells could promote the migration of colon cancer cells *in vitro* and *in vivo*, we further analyzed the specific regulatory mechanism used during this process. We determined the expression of RhoA/B/C in tumor cells before and after co-culturing with BMMCs by Western blotting. Figure 3A shows significantly increased expression levels of RhoA in CT26 cells after co-culturing with BMMCs for 24 h. However, the expression of RhoB and RhoC did not change significantly (data not shown).

We then evaluated the expression of vascular endothelial growth factor (VEGF) and TGF-β, which are key regulatory factors conducive to the invasion and angiogenesis of cancer. To assess the secretion of these cytokines by CT26 cells, we designed primers of TGF-β and VEGF A/B/C. The RNA of CT26 cells alone and CT26 cells co-cultured with BMMCs was extracted and the transcription level of the cytokines from the two groups was analyzed by Realtime-PCR. The comparison results show a significant increase of VEGF C in CT26 cells co-cultured with BMMCs for 16 h (*p* < 0.01) (Figure 3B). In addition, mast cells were also able to promote the expression of VEGF A and TGF-β in CT26 cells to a certain extent (*p* < 0.05) (Figure 3C).

### 2.4. MAPK/Rho-GTPase/STATs Pathways Participate in the Regulation of Mast Cell Function in Colon Cancer Development

**Figure 4:**
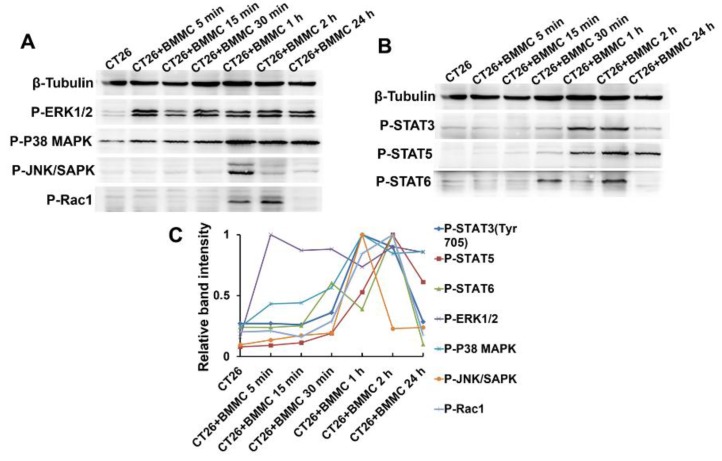
MAPK/Rho-GTPase/STATs pathways participate in the regulation mechanism of mast cells function to colon cancer cells. (**A**) CT26 cells were co-cultured with BMMCs for different time. Suspended BMMCs were then washed out and CT26 cells were further processed to cell lysates for western blotting analysis. Western blotting analysis detected the phosphorylation of signaling proteins in CT26 cells after BMMCs stimulation, including ERK 1/2, P38 MAPK, JNK/SAPK, and Rac1. (**B**) Western blotting analysis detected the phosphorylation of STATs pathway signaling protein in CT26 cells after BMMCs stimulation, including STAT3, STAT5 and STAT6. (**C**) Relative quantization of the pathways activation included Figure 4A,B during BMMCs stimulation. The band intensity was quantized by the Quantity One.

As one of the most important intracellular signal transduction pathways described above, MAPK pathway activation was evaluated in colon cancer cells during stimulation by mast cells. CT26 cells were co-cultured with BMMCs for 5 min, 15 min, 30 min, 1 h, 2 h and 24 h and all samples evaluated similarly. ERK 1/2 was extensively phosphorylated and reached peak activation at about 5 min. P38 MAPK was obviously phosphorylated with activation peaking at about one hour post-treatment. According to our results, both ERK and p38 MAPK continued to display high levels of phosphorylation in the CT26 cells after their peak activation. The phosphorylation of JNK in CT26 cells was not apparent until one hour post-treatment when it reached peak activation and was then rapidly degraded (Figure 4A,C).

Activation of Rho GTPase which can regulate cell migration and adhesion processes was also detected using the same method above. Results showed that CT26 cells co-cultured with mast cells exhibited appreciable Rac1 activation between one and two hours (Figure 4A,C). We did not detect appreciable activation of Cdc42 or other Rho GTPases (data not shown).

As members of STATs signaling pathways have been found to be involved in cytokine regulation of tumor cells, we systematically assessed STATs activation in tumor cells that have been in contact with mast cells. CT26 cells were co-cultured with mast cells by direct addition of BMMCs to the media for 5 min, 15 min, 30 min, 1 h, 2 h and 24 h. Whole cell lysates were then analyzed by Western blot to define the phosphorylation profiles of STATs activation. Within 30 min of BMMC treatment, STAT3 Y705 was extensively phosphorylated with activation peaking between one and two hours post-treatment. Furthermore, STAT5 and STAT6 were also phosphorylated with both reaching peak activation levels at about two hours post-treatment (Figure 4B,C). Other members of the STAT protein family did not show significant phosphorylation after mast cell stimulation of CT26 cells.

### 2.5. Targeted Fcε-PE40 Chimeric Toxin Can Specifically Induce BMMCs Apoptosis without Degranulation

**Figure 5:**
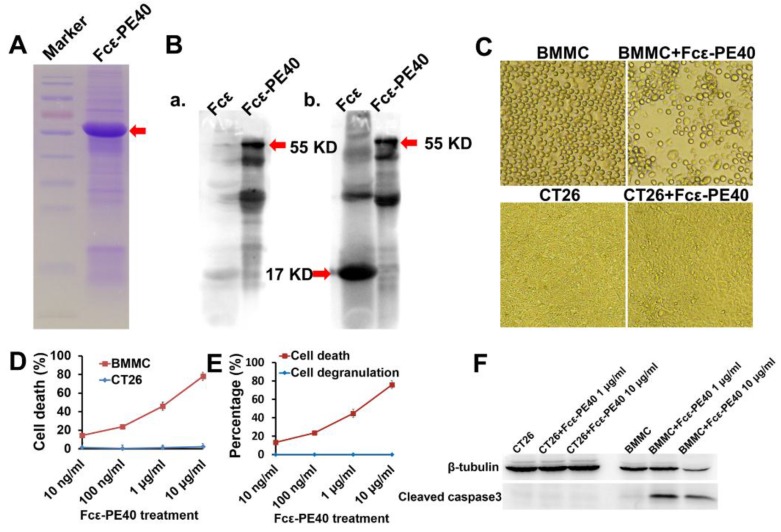
Mast cell targeted Fcε-PE40 chimeric toxin induce mast cells apoptosis without degranulation. (**A**) The purity and concentration of recombinant protein Fcε-PE40 chimeric toxin were measured by 12% SDS-PAGE (arrow indicating). (**B**) The protein Fcε (17 KD) and Fcε-PE40 chimeric toxin (55 KD) were verified by western blotting with Pseudomonas Exotoxin A antibody (a) and mouse IgE antibody (b). (**C**) Images show the growth of CT26 cells and BMMCs with or without Fcε-PE40 chimeric toxin (1 μg/mL) after 24 h. Magnification, ×100. Scale bar, 100 μm. (**D**) Cell death dose dependent curves after CT26 cells and BMMCs co-cultured with Fcε-PE40 for 24 h were assessed by MTS assay. (**E**) Mast cells’ degranulation after treated by Fcε-PE40 for 24 h was assessed by histamine release analysis. (**F**) Expression level of cleaved caspase3 in CT26 cells and BMMCs co-cultured with Fcε-PE40 for 24 h was detected by western blotting.

For the construction of the targeting chimeric protein, we used a fragment of the mouse IgE constant region (Fcε) that binds to mouse high-affinity IgE receptors. We used a sequence corresponding to amino acids 301–437, containing the C terminus of domain 2 and domain 3. The cDNA was cloned at the 5′ terminus of a truncated PE molecule (PE40) lacking the cell binding domain. The resulting plasmid pAF2302, encoding the Fcε-PE40 chimeric toxin, was characterized by restriction and sequence analysis (data not shown). Fcε-PE40 chimeric toxin was expressed in E. coli and characterized by gel electrophoresis (Figure 5A). Subcellular fractionation of expressing cells revealed that the insoluble fraction (inclusion bodies) was particularly enriched with the chimeric protein; the relative content was estimated to be 90%. This fraction was therefore the source of the chimeric protein used in our experiments. The protein was further characterized by Western blot analysis using antibodies against Pseudomonas Exotoxin A and mouse IgE (Figure 5B) and according to the molecular weight of the protein.

The effect of the chimeric protein was tested on BMMCs on the 42th day of their differentiation in culture (BMMC 77%). As shown in Figure 5C, Fcε-PE40 was cytotoxic to BMMC in a dose-dependent manner, with an ID50 of 1 μg/mL. At a high dose of chimeric protein, there was nearly 80% induction of BMMC death. On the contrary, Fcε-PE40 had no cytotoxic effect on CT26 cells (Figure 5C,D). Neither of the control proteins for either Fcε or PE40 was cytotoxic for BMMC (data not shown). We also tested whether binding of the Fcε-PE40 to its receptor on target cells triggered mast cell degranulation. Histamine release was again employed as an indicator of degranulation. No degranulation of mast cells was observed with any concentration of chimeric protein tested (Figure 5E).We further explored the mast cell death pathway induced by Fcε-PE40. Western Blotting results clearly show cleaved Caspase3 in the mast cell lysate after activation with Fcε-PE40 (Figure 5F), indicating that the Fcε-PE40 chimeric toxin depends on the caspase cascade to induce cell death. Thus, the Fcε-PE40 chimeric toxin can specifically target mast cells and induce caspase-dependent apoptosis without degranulation.

### 2.6. The Promotion of Mast Cell to Colon Cancer in Vivo Can Be Effectively Controlled by Targeted Fcε-PE40 Chimeric Toxin

**Figure 6:**
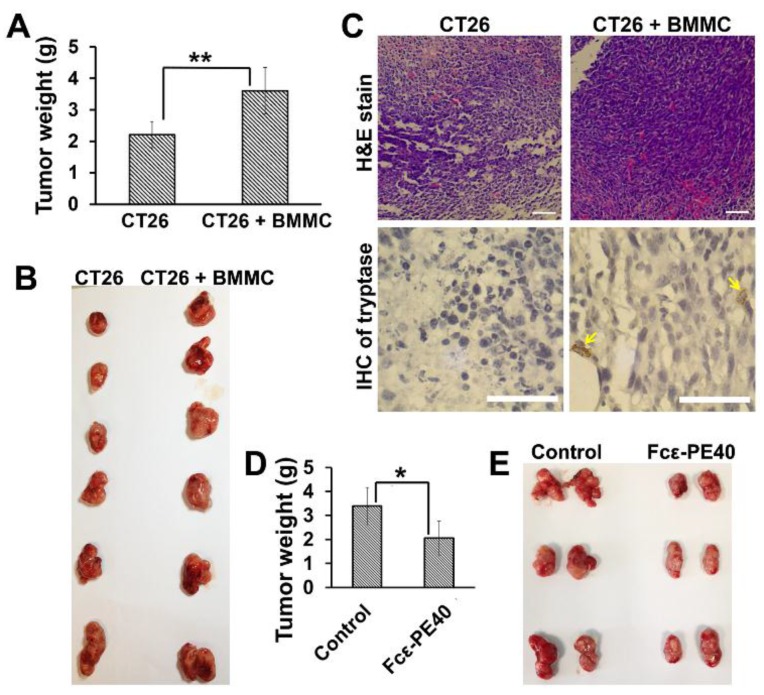
Mast cells targeted Fcε-PE40 chimeric toxin can assist colon cancer control *in vivo*. (**A**) Tumor growth in mice implanted with CT26 cells and CT26 cells together with BMMCs. Tumor growth is measured by the weight of solid tumor mass. (**B**) Solid tumor masses obtained from mice implanted with CT26 cells and CT26 cells together with BMMCs. (**C**) H & E staining and Immunohistochemistry of tryptase sections of tumor mass implanted with CT26 cells and CT26 cells together with BMMCs. Arrows indicate mast cells rich in tryptase in tumor tissues. Scale bar, 50 μm. (**D**) Determination of the tumor growth. Tumor weight was calculated 4 weeks after injection. (**E**) Representative image for tumor growth is shown. BAL B/C mice were subcutaneously injected with 2.0 × 10^6^ CT26 cells. Fcε-PE40 chimeric toxin was injected after 2 weeks. * *p* < 0.05; ** *p* < 0.01.

To determine the contribution of mast cells to tumorigenesis *in vivo*, CT26 cells alone and CT26 cells together with BMMCs (CT26 cells and BMMCs with the ratio of 20:1) were implanted into the flanks (2.0 × 10^6^ CT26 cells per flank) of BALB/C mice by subcutaneous injection. At four weeks post-injection, the mean weight and volume of xenograft tumors generated from group of CT26 cells alone was significantly less than those obtained from group of CT26 cells with BMMCs (*N* = 12 animals per group, *p* < 0.01) (Figure 6A). In addition, the shape of the tumor obtained from mice injected with CT26 cells plus BMMCs was more irregular than tumors from the control group, displaying branch-like growth and invasion and also showing severe adhesion with the surrounding tissue (Figure 6B). Meanwhile, H & E staining showed increased tumor cell density and abundant vascular distribution in xenografts and Immunohistochemistry staining of mast cell tryptase showed mast cells Infiltration in tissue from the group of CT26 cells plus BMMCs (Figure 6C). Based on the above findings, mast cells can significantly promote the proliferation and invasion of colon cancer cells *in vivo*.

To determine the contribution of targeted Fcε-PE40 chimeric protein to tumor control, we compared transplantation tumor growth in two groups of mice. CT26 cells were implanted by subcutaneous injection into one side of the flank (2.0 × 10^6^ CT26 cells per flank) of BALB/C mice. After two weeks, when subcutaneous tumor masses were apparent, one group had their tumor mass injected with Fcε-PE40 chimeric toxin. The control group had their tumors injected with the same volume of PBS. After two weeks of chimeric toxin injection, the mean weight and volume of tumor masses injected with Fcε-PE40 chimeric toxin were significantly smaller than those originating from the PBS-injected group (*N* = 12 animals per group, *p* < 0.01, Figure 6D). Compared to chimeric toxin injection group, tumor masses from control group display striking irregular shapes and adhesion with surrounding tissues (Figure 6E). Thus, we confirm that targeted Fcε-PE40 chimeric toxin can significantly contribute to the control of colon tumor development and can be considered an effective adjuvant therapy to colon cancer.

## 3. Discussion

Mast cells are not only an essential factor in allergy, but also play important role in inflammation. It is known that chronic inflammation plays a pivotal role in the initiation and progression of cancer [2,55]. However, mast cells may play a variety of roles in different local stromal conditions [39,40,45]. The mechanisms that mast cells use in the tumorigenesis of cancer are not totally clear. The dual roles of mast cells in both promoting and inhibiting tumor growth have been widely reported [41,42,43,44]; a high level of mast cell infiltration has been found to indicate a poor prognosis in skin cancer [56], prostate cancer [41], oral squamous cell carcinoma [57], and several types of lymphomas [58] but, on the contrary, high mast cell numbers usually indicate a good prognostic outcome for several other cancers such as ovarian cancer, breast cancer, and non-small cell lung cancer [59]. Summarizing recent research, mast cells can promote tumorigenesis in the following ways: promoting the proliferation of tumor cells by the secretion of growth factors; facilitating tumor cell detachment from the matrix and migration by secreting protease; providing angiogenic cytokines for angiogenesis in the local environment that leads to tumor progression. Detrimental activity on tumor growth can be attributed to cytotoxic cytokines and immune system regulation cytokines released from mast cells [60].

In this study, we show that mast cells play a positive role in the development and progression of colon cancer, the second highest type of cancer in Guangdong, China. We found that the “piecemeal degranulation” of mast cells could induce colon tumor cell proliferation and invasion *in vitro* and *in vivo*. The cytokines related with cell adhesion, cell dynamics and angiogenesis that are expressed in colon cancer cells were significantly changed in the presence of mast cells. Further study revealed that the above changes were triggered by the activation of the MAPK, Rho/ROCK and STATs pathways in colon cancer cells. These findings indicate that mast cells are essential for colon cancer progression and present a potential therapeutic target.

The current increasing incidence of tumors and research regarding cancer treatment over the past decades has led to a clear need for more effective immune therapy [61,62]. Toward this goal, we employed the Fcε-PE40 chimeric toxin, which represents a new approach to the adjuvant immunotherapy of colon cancer based on the targeted elimination of cells expressing FcεRI. We demonstrated that our Fcε-PE40 chimeric toxin is highly and specifically cytotoxic to mouse mast cells with a dose dependency *in vitro*. Using the subcutaneous colon cancer model in mice, we carried out three exponential increasing concentration levels of Fcε-PE40 chimeric toxin which were 10 μg, 100 μg and 1 mg while 100 μg and 1 mg Fcε-PE40 chimeric toxin could effectively inhibit colon cancer development *in vivo*. These results demonstrated that the effect of Fcε-PE40 chimeric toxin *in vivo* was also dose dependent. Currently we are establishing mice colon cancer models by AOM and AOM/DSS to imitate human colon cancer’s microenvironment to the greatest extent. The models will be very helpful to assess the effect of chimeric toxin through tail vein injection, intraperitoneal injection or through enema in the future study of cancer immune therapy.

A major concern regarding this new treatment is the possibility of anaphylaxis caused by the Fcε-PE40 chimeric toxin. Therefore, it is necessary to determine whether use of the chimeric protein could result in the release of allergic mediators by mast cells. We tested for the degranulation of mast cells after engagement with the chimeric toxin by measuring the release of histamine. There was no detectable histamine released which suggested that no degranulation occurred after the killing of mast cells by treatment with the chimeric protein. We further revealed that the chimeric protein induced mast cell death through caspase cascade dependent apoptosis, which does not lead to mast cell degranulation or the release of allergic mediators. It is the same cell death pathway utilized by other recombinant immunotoxins containing Pseudomonas Exotoxin A [63,64]. Thus, we have determined that the Fcε-PE40 chimeric toxin can kill mast cells by apoptosis without degranulation and inflammatory mediator release.

Currently, immunotherapy in the treatment of tumors is used to stimulate and enhance immune function and often functions as an adjunct therapy when combined with surgery, chemotherapy, radiotherapy and other conventional methods [65,66]. After eliminating the majority of the tumor by conventional methods, immune therapy was used to eradicate any remaining tumor cells, which can improve overall efficacy in the treatment of cancer. With the now known association between inflammation and cancer, more and more research has focused on the control of inflammation to prevent the development and progression of cancer. As one of the important inflammatory cells, mast cells have been increasingly considered as a immunotherapy target in recent years [67]. The Fcε-PE40 chimeric toxin we constructed can specifically kill mast cells around tumor cells without damage to other immune cells and can help to block the spreading and proliferation of cancer. Importantly, this process does not cause the degranulation of mast cells or trigger an allergic reaction. In addition, because mast cells are a key player in allergic reactions and other inflammatory diseases, this chimeric protein might also be used in the treatment of allergic and inflammatory diseases.

In addition to the mouse mast cell Fcε receptor targeted chimeric protein, we also constructed a chimeric protein targeting the human mast cell Fcε receptor, whose biologic activity has also been tested and verified *in vitro*. However, at present there are still restrictions on the use of this chimeric protein *in vivo*. Because it contains exogenous antigens, prolonged administration of the chimeric protein can result in the generation of antibodies that can block and reduce its effect. At the same time, the chimeric protein could also be an allergen and induce an allergic reaction *in vivo*. In order to avoid adverse reactions and optimize its effects *in vivo*, we have tried to modify the chimeric protein through bioinformatics and molecular biology. We have analyzed the immunogenic and allergenic epitopes by bioinformatics. The Fcε-PE40 chimeric toxin then was transformed through point mutations according to the results of the above analysis in order to reduce its immunogenicity and allergenicity without affecting its biological activity. The above work is ongoing at present. Successfully obtaining chimeric proteins with both low antigenicity and low allergenicity will provide more security for immunotherapy targeting mast cells in the treatment of cancer, allergies and inflammatory diseases.

## 4. Conclusions

We confirmed that mast cells could serve a positive function in the development of colon cancer by promoting cancer cell invasion and angiogenesis through MAPK/Rho-GTPase/STATs pathways. Additionally, we also revealed the effective function of mast-cell-targeted chimeric toxin, which could kill mast cells to help control the development of colon cancer without inducing mast cell degranulation and anaphylactic reaction. Thus, mast-cell-targeted chimeric toxin may be developed as an adjunctive therapy in colon cancer treatment.

## 5. Materials and Methods

### 5.1. Patients and Tissues

Paraffin-embedded specimens of colonic polyps from 18 patients and colonic adenocarcinoma tissue from 35 patients who had surgery were studied. In addition, specimens of colonic adenocarcinoma were divided into either well-differentiated (*N* = 20) or poorly-differentiated groups (*N* = 15) according to pathological grading. Depending on whether there were lymph nodes or distant metastases of cancer cells, colonic adenocarcinoma specimens were further divided into two groups of either non-metastatic or metastatic colorectal cancer. All of the specimens were obtained from the Second Affiliated Hospital of Guangzhou Medical University (Guangzhou, Guangdong) and all procedures were approved by the ethics committee of the Second Affiliated Hospital of Guangzhou Medical University.

### 5.2. Mice and Cell Culture

BALB/c (6-weeks old) mice were obtained from the Experimental Animal Center of Guangdong Province. Mice were maintained under specific pathogen-free conditions. 2 × 10^6^ CT26 cells were injected under the skin of each mouse (control group, *N* = 12). CT26 cells and bone marrow-derived mast cells (BMMC) mixed together at a 20:1 ratio were injected in the same manner (experimental group, *N* = 12). In another experiment, after 2 weeks of tumor development, Fcε-PE40 chimeric toxin (100 μg) was injected into the tumor mass of each mouse.

CT26 cells were purchased from the Chinese Academy of Sciences Cell Bank and grown in Dulbecco’s Modified Eagle Medium (DMEM) supplemented with 10% FBS (GIBCO, Auckland, New Zealand). Bone marrow-derived mast cells were prepared from bone marrow cells obtained from mouse femurs and cultured in 1640 Medium supplemented with 10% FBS (GIBCO), 100 ng/mL IL-3 (Peprotech, Rocky Hill, NJ, USA) and 100 ng/mL SCF (Peprotech) for more than 4 weeks to induce mast cell differentiation.

All animal protocols and procedures were conducted under the approval of the Ethical Committee of Guangdong Provincial Animal Experiment Center. All animals received human care and that study protocols comply with the institution’s guidelines.

### 5.3. Immunohistochemistry and MC Counting

Immunohistochemistry studies to quantify the number of mast cells in tumors were conducted on the patient specimens using antibodies against c-kit and tryptase (Abcam, Cambridge, MA, USA), which are specifically expressed in mast cells [43,44]. 4 µm sections of the human colon tissues were treated for antigen retrieval for one hour in a steamer containing 10 mmol/L citrate buffer (pH 6.0). These tissues were then immersed in methanol containing 0.3% hydrogen peroxidase for 20 min to block endogenous peroxidase activity followed by incubation in 2.5% blocking serum to reduce nonspecific binding. The sections were then incubated for one hour at 37 °C with anti-CD117 and anti-tryptase (Abcam, Cambridge, MA, USA) at a concentration of 1:100 dilution. Avidin-biotin counterstaining was done according to the manufacturer’s recommendations (Maixin, Fuzhou, China). Vector Red was used as a chromogen, and hematoxylin was used for counterstaining.

Toluidine blue (Sigma-Aldrich Corp., St. Louis, MO, USA) staining (pH 2.0–2.5) for MCs was carried out on smeared slides according to the method previously described [50].

The quality and acceptability of the stained samples was independently evaluated by two gastrointestinal pathologists. The percentage of positive cells was quantitatively determined in each sample and pictures were taken with the EVOS XL-Core Image Analysis System (AMG, Bothell, WA, USA). We got the mast cell staining index scores by counting cells with tryptase positive frequency per 10 high-power fields investigated by two pathologists.

### 5.4. Plasmid Construction, Protein Expression and Partial Purification

The mouse IgE Fcε-PE40 chimeric toxin, with an N-terminal Strep II tag and a C-terminal endoplasmic reticulum retention signal (KDEL), was synthesized by Biosune Biotechnology Co Ltd. By using a pair of primers (5′ CCGCTCGAGTTATTAAAGTTCATCCTTTGGTGGC 3′, and (5′ GGAATTCCATATGTGGTCTCACCCACAATTCGAAAAAGGA 3′), the chimeric toxin was amplified and linked into the pET21b vector at Nde I/Xho I sites (Ampicillin resistant; Novagen, GER), followed by frame-confirmation by sequencing analysis. The chimeric protein was expressed in *Escherichia coli* strain Rosetta. Expression induction was performed for 4 h in the presence of isopropyl beta-d-thiogalactoside (IPTG, 1 mM final concentration). Bacteria were harvested by centrifugation and lysed by sonication in binding buffer (150 mM NaCl, 100 mM Tris pH 8.0 and 1 mM EDTA). The homogenate then was centrifuged at 10,000× *g* for 30 min at 4 °C. The supernatant (soluble fraction) was removed and kept for SDS-PAGE analysis; the pellet was denatured in urea buffer (6 M urea, 150 mM NaCl, 100 mM Tris pH 8.0 and 1 mM EDTA) and stirred overnight at 4 °C. The solubilized protein was subjected to gradient dialysis with refolding buffer (binding buffer containing 4, 2, 1, 0.5, and 0 M urea). The refolded protein solution was dialyzed against PBS at 4 °C, centrifuged at 10,000× *g* for 30 min and the aliquots of the resulting supernatant were kept at −20 °C until use.

### 5.5. Immunoblotting

Whole proteins from the cultured cells were extracted according to the manufacturer’s protocol (RIPA) (Biyuntian, Shanghai, China), followed by concentration investigation. All samples were separated by SDS-PAGE and transferred following standard protocols. Antibodies to CD117 (Abcam, Cambridge, MA, USA) and FcεRI (Abcam) were used to identify mature mast cells. Rho-GTPase Antibody Sampler Kit (Cell Signaling Technology, Danvers, MA, USA), Phospho-MAPK Family Antibody Sampler Kit (Cell Signaling Technology) and Phospho-Stat Antibody Sampler Kit (Cell Signaling Technology), were used to detect the activation of signaling pathways in colon tumor cells. Antibody to cleaved caspase3 (Millipore, Temecula, CA, USA) was used to identify cell apoptosis. Antibody to β-tubulin (Cell Signaling Technology) was used to ensure the consistency of each cell lysate. Antibody to *Pseudomonas* Exotoxin A (Sigma-Aldrich Corp., St. Louis, MO, USA) and mouse anti-Human IgE antibody (Abcam) were used to identify the recombinant protein toxin. HRP-conjugated secondary antibodies were purchased from Cell Signaling and SuperSignal chemiluminescent reagent was purchased from Thermo Fisher Scientific (Waltham, MA, USA).

Protein expression was evaluated using Quantity One software according to immunoblotting band intensity.

### 5.6. Flow Cytometry

Mast cells obtained during different induction times were stained with fluorescence-labeled anti-CD117 antibody or its isotype control antibody. Positive cells were detected by flow cytometry.

### 5.7. Histamine Release

Mast cell challenge assay was conducted according to the protocol previously described [51]. Briefly, 3000 induced mature mast cells were plated into the wells of the ELISA plate with each well containing glass fiber at the bottom. Mast cells in plates were then treated with different concentrations of calcium ionophore (A23187) (Sigma-Aldrich) and Fcε-PE40 chimeric toxin. Mast cells treated with 0.5% Triton-100 [XT] were considered to contain the maximum (MAX) histamine release. Culture medium alone was used as the base value. Mast cells without any treatment were considered to demonstrate the natural release of histamine and represented the “piecemeal degranulation” of mast cells [26,27]. Plates with mast cells were incubated at 37 °C for two hours and then washed to eliminate cells and cell debris. At that time, histamine released from the mast cells had already been captured by the glass fiber at the bottom of the wells. A coupling reagent with a fluorescent probe that can bind to histamine was added to the wells. Finally, the plate was read using a histamine release analysis system: Hisreader501 (REFLAB, R-Biopharm, Darmstadt, Germany). HisReader501 assigns a relative value to each well in the plate according to its fluorescence intensity. Percent histamine release = (Experimental well value-base value)/(MAX release-base value) × 100%.

### 5.8. Cell Proliferation Assay

CT26 cells were plated 5 × 10^3^ cells/well into 96-well plates. BMMCs were then plated into co-culture group wells with the ratio of 1:5 to CT26 cells. After 48 h in culture, cell proliferation was assessed using CellTiter 96 Aqueous MTS (3-[4,5-dimethyl-thiazol-2-yl] -5-[3-carboxymethoxyphenyl]-2-[4-sulfophenyl]-2H-tetrazo-lium, inner salt) assay (Promega, Madison, WI, USA) according to the manufacturer’s instructions. Briefly, reagent was added to each well and incubated at 37 °C for three hours. Cell proliferation was assessed by measuring the absorbance at 490 nm with a microplate reader (Thermo Fisher Scientific).

### 5.9. Colon Cancer Epithelial Cell Invasion Assay

CT26 cells were seeded into 6-well plates at 3 × 10^5^ per well. BMMCs were then plated into co-culture group wells with the ratio of 1:5 to CT26 cells. After cell adherence, a fixed width of the cell monolayer was scratched off using a pipette tip. Movement of CT26 cells into a wound scratched [52] into the monolayer with or without added BMMCs was then monitored with a phase contrast 20× objective using an inverted microscope (AMG, EVOS XL-Core, Seattle, DC, USA). Images were obtained at 24 and 48 h of culture.

### 5.10. RNA Extraction and Quantitative Real-Time PCR

Total RNA was extracted from CT26 cells using RNeasy Mini Kit (Qiagen, Dusseldorf, Germany). cDNAs were synthesized using PrimeScript™ RT reagent Kit (Takara, Dalian, China) and real-time PCR was performed using SYBR® Select Master Mix (Applied Biosystems, Foster, CA, USA) on an ABI 7500 series PCR machine (Applied Biosystems). Primer pairs (Table 1) were used to detect target gene transcripts.

The PCR mixture used for quantitative cDNA amplification contained 1 μL cDNA (1:10 dilution), 5 μL SYBR Green Mix (Applied Biosystems), 0.3 μL of each primer (sense and antisense, 10 μM) and 3.4 μL nuclease-free water. The reaction mixture was heated to 50 °C for two minutes, 95 °C for two minutes, and cycled (15 s at 95 °C; 31 s at 60 °C) for 40 cycles. Fluorescent data for quantification was collected at 72 °C. Melting curve analysis was performed over a range of 60–95 °C in order to verify single product amplification at the end of the assay. All samples were tested in triplicate and the amount of mRNA detected was normalized to GAPDH transcription level.

### 5.11. Statistical Analysis

All experiments were repeated three times. Results are expressed as the means ± SD, unless differently indicated. Comparisons between two groups were carried out with the 2-tailed Student *t* test for unpaired samples. In all tests, a value of *p* < 0.05 was considered significant.

## Figures and Tables

**Table 1:** Primer pairs used for target gene transcripts.

Target Genes	Sequences (5′ → 3′)	Amplified Fragment Length
*VEGF-a*	GAGAGCAGAAGTCCCATGAAGTGA	185 bp
	GCACTCCAGGGCTTCATCGTTA	
*VEGF-b*	CAGAAGAAAGTGGTGCCATGGAT	177 bp
	CACACATTCCAGGCCATCGTC	
*VEGF-c*	CTCACAAGGCCCCAAACCAG	145 bp
	GTTAGCTGCCTGACACTGTGGTAAT	
*TGF-β*	GGCTGAACCAAGGAGACGGAAT	145 bp
	CGGTTCATGTCATGGATGGTGC	
*GAPDH*	GTGGAAGGGCTCATGACCACA	188 bp
	AAGGCCATGCCAGTGAGCTTC	

## Abbreviations

The following abbreviations are used in this manuscript:

PEA	Pseudomonsexotoxin A
BMMC	Bone marrow mast cell
